# Preoperative sport improves the outcome of lumbar disc surgery: a prospective monocentric cohort study

**DOI:** 10.1007/s10143-017-0811-6

**Published:** 2017-01-13

**Authors:** Anja Tschugg, Sara Lener, Sebastian Hartmann, Matthias Wildauer, Wolfgang N. Löscher, Sabrina Neururer, Claudius Thomé

**Affiliations:** 1 Department of Neurosurgery, Innsbruck Medical University, Anichstr. 35, A-6020 Innsbruck, Austria; 2 Department of Neuroradiology, Innsbruck Medical University, Innsbruck, Austria; 3 Department of Neurology, Innsbruck Medical University, Innsbruck, Austria; 4 Department of Medical Statistics and Health Economics, Innsbruck Medical University, Innsbruck, Austria

**Keywords:** Radiculopathy, Lumbar sequestrectomy, Disc herniation, Improvement after disc herniation, Physical activity, Sports

## Abstract

A lumbar disc herniation resulting in surgery may be an incisive event in a patient’s everyday life. The patient’s recovery after sequestrectomy may be influenced by several factors. There is evidence that regular physical activity can lower pain perception and improve the outcome after surgery. For this purpose, we hypothesized that patients performing regular sports prior to lumbar disc surgery might have less pain perception and disability thereafter. Fifty-two participants with a single lumbar disc herniation confirmed on MRI treated by a lumbar sequestrectomy were included in the trial. They were categorized into two groups based on their self-reported level of physical activity prior to surgery: group NS, no regular physical activity and group S, with regular physical activity. Further evaluation included a detailed medical history, a physical examination, and various questionnaires: Visual Analog Scale (VAS), Beck-Depression-Inventory (BDI), Oswestry Disability Index (ODI), Core Outcome Measure Index (COMI), and the EuroQoL-5Dimension (EQ- 5D). Surgery had an excellent overall improvement of pain and disability (*p* < 0.005). The ODI, COMI, and EQ-5D differed 6 months after intervention (*p* < 0.05) favoring the sports group. Leg and back pain on VAS was also significantly less in group B than in group A, 12 months after surgery (*p* < 0.05). Preoperative regular physical activity is an important influencing factor for the overall satisfaction and disability after lumbar disc surgery. The importance of sports may have been underestimated for surgical outcomes.

## Introduction

A lumbar disc herniation is considered a major source of low back pain and radiculopathy by mechanical compression of the corresponding lumbar nerve root. The annual costs in terms of lost productivity, medical expenses, and worker’s compensation benefits are significant. [1] A minority of patients affected require surgical treatment by lumbar sequestrectomy, especially in medical refractory pain or in the presence of neurological deficits. [2, 3]

A lumbar disc herniation prompting surgery may be an incisive event in a patient’s everyday life. The patient’s recovery and improvement of pain after lumbar spine surgery may be influenced by several factors. [4] It is known that a physically active lifestyle is crucial for a variety of health-related benefits. It has also been suggested that regular physical activity can lower pain perception and improve outcome after surgery. [5] In contrast, reduced exercise has been linked to various chronic health disorders particularly to musculoskeletal complaints. [6] Physical activity in the management of low back pain is therefore widely recommended. [7]

Nevertheless, the influence of regular physical activity prior to lumbar spine surgery on postoperative outcome has not yet been investigated in a prospective clinical trial. Thus, we hypothesized that patients who perform sports regularly before lumbar disc surgery might have less pain and disability thereafter. Therefore, the objective of this study was to prospectively assess the effect of baseline physical activity level on pain, satisfaction, and disability after lumbar disc surgery.

## Material and methods

### Subjects

The prospective study was purely observational, and there were no recommendations for additional diagnostic measures or interventions. Pain management was not delayed or altered by participation in this study. All subjects gave their informed consent. The study was approved by the Local Ethics Committee in accordance with the ethical principles originating from the Declaration of Helsinki and in compliance with Good Clinical Practice. Consecutive patients were considered for inclusion, if they had a single-level disc herniation confirmed on magnetic resonance imaging (MRI). All patients had an indication for sequestrectomy according to the guidelines of the German Society of Neurosurgery (DGNC) and the German Society of Orthopedics and Orthopedic Surgery (DGOOC). All participants were on best medical pain treatment, but sufficient pain relief was not achieved. No previous back surgery had been performed in any of the patients. None of the included patients had a history of peripheral nervous system disorders like metabolic or toxic damage of peripheral nerves. The patients were categorized into two groups based on their self-reported level of physical activity prior to surgery: group NS, no regular physical activity and group S, with regular daily to weekly physical activity. The type of sport was also documented and classified according to the classification of the American Heart Association and American College of Cardiology. This classification is based on the dynamic and static intensity: dynamic component: A (low) <50%, B (moderate) 50–75%, and C (high) >75%; static component: I (low) <10%, II (moderate) 10–20%, and III (high) >30%. [8] According to our local standard of care, all patients received a postoperative rehabilitation program starting 4 weeks after sequestrectomy, which consisted of eight sessions lasting 45 min each.

### Questionnaires, medical history, and clinical examination

The prospectively planned evaluation included a detailed medical history, a physical examination, and various questionnaires. All data were recorded the day before surgery, within 1 week, 6 and 12 months after surgery. In this preliminary cohort study, the VAS for back pain 1-year post intervention was set as the primary outcome parameter, being fully aware, that under certain circumstances, the planned number of patients might not result in robust indicators for standard deviations and group differences. The VAS was determined for leg and back pain separately. The Beck Depression Inventory (BDI) is a multiple-choice self-reported inventory for measuring the severity of depression and responsiveness to treatment. [9] The degree of disability was assessed with the Oswestry Disability Index (ODI), which is divided into ten items designed to assess multiple aspects of disability with respect to pain. [10] The Core Outcome Measure Index (COMI) comprises a series of questions covering the domains of pain, back specific function, work disability, and patient’s satisfaction. [11] Furthermore, the generic health status is assessed with the EuroQoL-5Dimension (EQ-5D). [12] Neurological status and the quality and quantity of current pain medication in accordance to the WHO guidelines for pain treatment, including nerve root and facet joint injections, were documented.

### Magnetic resonance imaging

Preoperative Magnetic Resonance Imaging (MRI) of the lumbar spine was performed in a standardized fashion on a 3.0-T MRI scanner (Siemens, Verio). The protocols included sagittal T1-TSE and T2-TSE, axial T1-TSE and PD/T2-TSE. Each MRI was examined for the evidence of disc degeneration (Pfirrmann degeneration grade) and degenerative changes of the intervertebral endplates (Modic changes) by an independent neuroradiologist, blinded to the clinical signs and symptoms. [13, 14] Furthermore, the quantitative measurements of multifidus and erector spinae muscles were taken from T2-weighted axial images using ImagJ imaging software (version 1.51, National Institutes of Health, Bethesda, Maryland). The muscle measurements were obtained bilaterally at the level of spinous process of L4 and included the following: total muscle size (cm^2^) and fat-free area (%). [15]

### Surgical procedures

Surgery was performed after induction of general endotracheal anesthesia and with the assistance of an operating microscope (Pentero, Carl Zeiss Co.) while the patient was in a prone position, by two surgeons in a standardized manner. The spinal canal harboring the sequestrated disc material was exposed by performing a minimal interlaminar fenestration. Based on results of previous trials, only the herniated material was removed and the herniated space was not entered. [3] Intraoperative problems such as surgery-related complications and postoperative complications like re-operations, recurrent disc herniations, infection, or bleeding were recorded, and these patients were excluded from further analyses to minimize heterogeneity of the cohort.

### Statistical analysis

All values were expressed as mean ± SD. The Kolmogorov–Smirnov test was used for testing normal distribution. The unpaired Student’s *t* test, Mann–Whitney U test, and Fisher’s exact test were used to analyze differences in clinical and demographic characteristics and in clinical outcome variables. A *p* value <0.05 was considered statistically significant. All statistical evaluations were performed with SPSS Version 21.0 (IBM Corp. Released 2012. IBM SPSS Statistics for Windows, Version 21.0, NY: IBM Corp.). Figures were designed using GraphPad Prism (version 5.0 for Mac OS X, GraphPad Software, La Jolla California USA, www.graphpad.com).

## Results

Fifty-two consecutive patients met the inclusion criteria and could be enrolled in the trial. By chance, they were divided symmetrically into patients with regular physical activity (group S; *n* = 26) and those without (group NS; *n* = 26). The loss to postoperative 6-month follow-up was 1.9% and to 12-month follow-up was 3.8%. A recurrent disc herniation occurred in six (23%) patients in group NS and in three (11.5%) patients in group S (*p* = 0.465). An accidental durotomy occurred in one patient in group NS. These patients were excluded from further statistical analysis, as these factors could have influenced the outcome data.

**Fig. 1:**
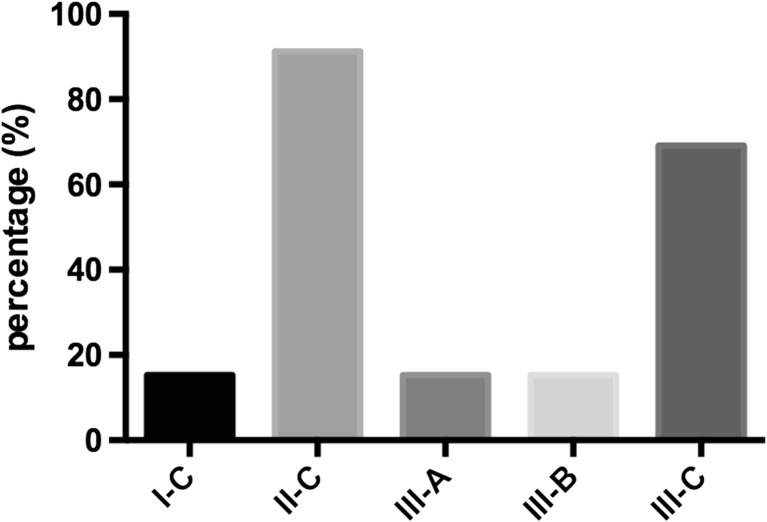
Type and distribution of physical activity. The type of sport was classified according to the classification of the American Heart Association and American College of Cardiology. This classification is based on the dynamic and static intensity: dynamic component: **a** (low) <50%, **b** (moderate) 50–75%, **c** (high) >75%; static component: *I* (low) <10%, *II* (moderate) 10–20%, *III* (high) >30%. [8]

The preoperative demographic data is described in Table 1. The most common Pfirrmann grade was IV° in both groups and there was not statistically significant difference between groups. 80.7% of group NS and 46.1% of group S showed Modic changes in MRI (*p* = 0.006). There were no significant differences in total muscle size or fatty degeneration (*p* > 0.05). Study participants preferred sports with high dynamic and moderate intensity (see Fig. 1).

**Table 1:** Demographic characteristics of patients with lumbar disc herniation

Demographic characteristics		NS	S
Mean age, years (SD)		44 (±11)	44 (±10)
Female/male ratio		11/15	10/16
Mean BMI (SD)		27 (±4)	26 (±3)
Smoking, *n* (%)		17/26 (65)	12/26 (46)
Cigarettes per day (SD)		9 (±9)	5 (±8)
Alcohol	None, *n* (%)	5/26 (19)	8/26 (30)
	Weekly, *n* (%)	2/26 (7)	0/32 (0)
	Incidentally, *n* (%)	19/26 (73)	18/26 (69)
ASA score	1, *n* (%)	14/26 (53)	17/26 (65)
	2, *n* (%)	12/26 (46)	9/26 (34)
Nerve root injection with steroid, *n* (%)		6/26 (23)	5/26 (19)
Mean duration of pain in days (SD)		130 ± 288	182 ± 230
Leg-raising test	Positive, *n* (%)	20/26 (76)	22/26 (84)
Radicular pain	L3, *n* (%)	3/26 (11)	1/26 (3)
	L4, *n* (%)	2/26 (7)	3/26 (11)
	L5, *n* (%)	12/26 (46)	8/26 (30)
	S1, *n* (%)	9/26 (34)	14/26 (53)
Recurrent disk herniation		6/26 (23)	3/26 (11)
Modic changes	None, *n* (%)	5/26 (19)	14/26 (53)
	Type 1, *n* (%)	0/26 (0)	2/26 (7)
	Type 2, *n* (%)	20/26 (76)	9/26 (34)
	Type 3, *n* (%)	1/26 (3)	1/26 (3)
Pfirrmann classification	Grade III, n (%)	5/26 (19)	5/26 (19)
	Grade IV, n (%)	16/25 (61)	18/26 (69)
	Grade V, n (%)	5/26(19)	3/26 (11)
Paraspinal muscle	Total muscle size, cm^2^	55 ± 9	55 ± 10
	Fat-free area, (%)	83 ± 6	84 ± 5

Group *NS* patients without regular sports prior to surgery, group *S* patients with regular sports prior to surgery, *ASA* American Society of Anesthesiology, *BMI* body mass index, *n* number of patients, *SD* standard deviation

Analysis of BDI demonstrated no significant intergroup differences pre- and postoperatively (*p* > 0.05).

EQ-5D index, COMI, and ODI showed a remarkable increase in the quality of life, overall outcome, and disability 12 months after lumbar sequestrectomy in both groups (*p* < 0.005) (Table 2).

**Table 2:** Pre and postoperative differences in multidimensional scores

Questionnaire	Pre			Pre 1 week		1 week			1 week –6 months		6 months			6–12 months		12 months			pre 12 months	
	NS	S	p⊥	NS p⨍	S p⨍	NS	S	p⊥	NS p⨍	S p⨍	NS	S	p⊥	NS p⨍	S p⨍	NS	S	p⊥	NS p⨍	S p⨍
ODI sum	38.3 ± 17	36.5 ± 17	n.s.	*0.024*	*0.013*	26.8 ± 18	25.3 ± 15	n.s.	*0.001*	*0.000*	11.9 ± 11	5.0 ± 6	*0.025*	n.s.	n.s.	11.1 ± 10	6.0 ± 8	n.s.	*0.000*	*0.000*
Pain intensity	2.5 ± 1	2.5 ± 1	n.s.	*0.001*	*0.000*	1.0 ± 0	1.0 ± 0	n.s.	n.s.	n.s.	1.0 ± 0.8	0.5 ± 08	*0.032*	n.s.	n.s.	0.8 ± 0	0.6 ± 0	n.s.	*0.001*	*0.000*
Personal care	0.8 ± 0	0.9 ± 0	n.s.	n.s.	*0.047*	0.5 ± 0	0.6 ± 1	n.s.	n.s.	*0.047*	0.0 ± 0	0.0 ± 0	n.s.	n.s.	n.s.	0.1 ± 0	0.0 ± 0	n.s.	*0.007*	*0.001*
Lifting	2.4 ± 1	2.0 ± 1	n.s.	n.s.	n.s.	2.8 ± 1	2.2 ± 1	n.s.	*0.000*	*0.001*	1.0 ± 1	0.3 ± 0	*0.005*	*0.002*	*0.007*	0.9 ± 0	0.8 ± 0	n.s.	*0.000*	*0.001*
Walking	1.5 ± 1	1.3 ± 1	n.s.	n.s	n.s.	1.1 ± 1	1.0 ± 0	n.s.	*0.008*	*0.001*	0.2 ± 0	0.0 ± 0	*0.034*	n.s.	n.s.	0.2 ± 0	0.1 ± 0	n.s.	*0.001*	*0.000*
Sitting	2.1 ± 1	2.0 ± 1	n.s.	n.s.	n.s.	1.7 ± 1	1.6 ± 1	n.s.	n.s.	*0.003*	0.9 ± 1	0.5 ± 0	n.s	n.s.	n.s.	0.8 ± 0	0.3 ± 0	n.s.	*0.002*	*0.001*
Standing	2.2 ± 1	2.5 ± 1	n.s.	n.s.	*0.013*	1.7 ± 1	1.6 ± 1	n.s.	*0.009*	*0.003*	0.8 ± 1	0.3 ± 0	*0.049*	n.s.	n.s.	0.8 ± 0	0.4 ± 0	n.s.	*0.001*	*0.000*
Sleeping	1.4 ± 1	1.3 ± 1	n.s.	*0.031*	*0.002*	0.7 ± 0	0.7 ± 0	n.s.	n.s.	*0.008*	0.5 ± 0	0.3 ± 0	n.s.	n.s.	n.s.	0.7 ± 0	0.3 ± 0	n.s.	*0.028*	*0.000*
Sex life	1.6 ± 1	1.6 ± 1	n.s.	n.s.	*0.036*	1.2 ± 1	0.9 ± 1	n.s.	*0.002*	n.s.	0.2 ± 0	0.1 ± 0	n.s.	n.s.	n.s.	0.1 ± 0	0.0 ± 0	n.s.	*0.002*	*0.002*
Social life	1.8 ± 1	2.0 ± 1	n.s.	*0.001*	*0.002*	0.6 ± 1	1.0 ± 1	n.s.	n.s.	*0.016*	0.4 ± 0	0.1 ± 0	n.s.	n.s.	n.s.	0.4 ± 1	0.0 ± 0	n.s.	*0.004*	*0.000*
Traveling	1.7 ± 1	2.1 ± **1**	n.s.	n.s.	*0.046*	1.2 ± 1	1.3 ± 1	n.s.	n.s.	*0.001*	0.5 ± 1	0.1 ± 0	n.s.	n.s.	n.s	0.3 ± 0	0.0 ± 0	n.s.	*0.016*	*0.000*
COMI	6.5 ± 1	6.4 ± 1	n.s.	*0.000*	*0.000*	4.8 ± 1	4.3 ± 1	n.s.	*0.000*	*0.000*	1.8 ± 1	0.9 ± 0	*0.043*	n.s.	n.s.	1.4 ± 1	0.7 ± 1	*0.025*	*0.000*	*0.000*
EQ-5D	0.82 ± 0	0.84 ± 0	n.s.	*0.004*	n.s.	0.89 ± 0	0.88 ± 0	n.s.	n.s.	0.000	0.93 ± 0	0.96 ± 0	*0.029*	n.s.	n.s.	0.95 ± 0	0.96 ± 0	n.s.	*0.000*	*0.000*

Group *NS* patients without regular physical activity prior to surgery, group *S* patients with regular physical activity prior to surgery. Data is presented as mean ± SD. A *p* value < 0.05 was considered statistically significant. *COMI* Core Outcome Measure Index, *EQ-5D* Euro-Quality of Life-5Dimension, *ODI* Oswestry Disability Index, *p* ***⊥*** differences between groups, *p⨍* differences in follow-up [10–12]

The overall ODI differed 6 months postoperatively: group NS 11.9 ± 11 vs. group S 5.0 ± 6 (*p* < 0.05). Differences were found particularly in pain intensity (group NS: 1.0 ± 0.8 vs. group S: 0.5 ± 0), lifting (group NS: 1.0 ± 0 vs. group S: 0.3 ± 0), walking (group NS: 0.2 ± 0 vs. group S: 0.0 ± 0), and standing (group NS: 0.8 ± 1 vs. group S: 0.3 ± 0) (*p* < 0.05). Group S showed a significantly higher quality of life 6 months postoperatively, group NS 0.93 ± 0 vs. group S 0.96 ± 0, respectively (*p* < 0.05). COMI also revealed differences between the two groups 6 and 12 months postoperatively (*p* < 0.05).

**Fig. 2:**
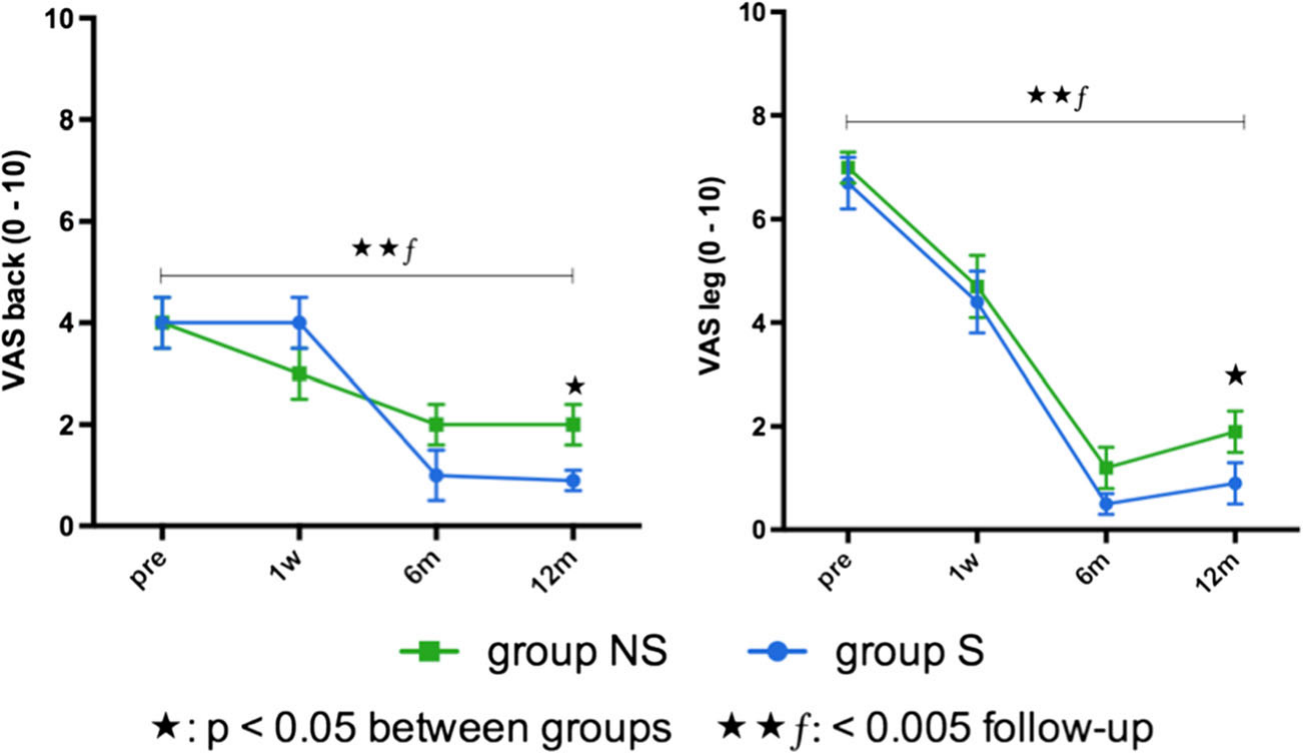
Differences in VAS for leg and back pain 1 year after surgery. Leg and back pain on VAS was rated significantly higher in group NS (without regular sports) than in group S (with regular sports) 12 months postoperatively. *Pre* preoperative, *1 w* 1 week, *6 m* 6 months, *12 m* 12 months. Data is presented as mean and standard deviation. ✱*p* < 0.05 (statistical significant), ✱✱*p* < 0.005 (highly statistical significant), *⨍*, follow-up

VAS for leg and back pain improved significantly in both groups after 12 months (*p* < 0.005). Leg and back pain on VAS was rated significantly lower in group S than in group NS 12 months postoperatively (*p* < 0.05, Fig. 2). Preoperative motor deficits improved significantly in both groups after 12-month follow-up (*p* ≤ 0.005). Seventy percent in the NS group and 62% in the S group took analgesics on a regular basis before surgery (Table 3). No permanent pain medication was used postoperatively.

**Table 3:** Preoperative pain medication

	NS	S
	all	all
No medication, *n* (%)	8/26 (30)	10/26 (38)
Non-opioid analgetics, *n* (%)	14/26 (53)	12/26 (45)
Naproxene mg/d	2000	2000
Metamizol mg/d	6000	5500
Paracetamol mg/d	4500	3500
Diclofenac mg/d	500	300
Dexibuprofen mg/d	4000	3600
weak opioid analgetics, *n* (%)	3/26 (11)	7/26 (26)
Tramadol mg/d	550	850
Strong opioid analgetics, *n* (%)	4/26 (15)	5/26 (19)
Oxycodon mg/d	40	25
Piritramid mg/d	22.5	22.5

70% in the NS group and 62% in the S group took analgesics on a regular basis before surgery. *n* number of patients, *NS* group without regular sports, *S* group with regular sports

## Discussion

The authors present the first prospective clinical trial investigating the influence of preoperative sports on the patient’s satisfaction and recovery after lumbar sequestrectomy. Disc surgery resulted in an overall significant improvement after 12-month follow-up, whereas patients doing preoperative sports on a regular basis showed a significantly better outcome. Leg and back pain 1 year after surgery was worse in the group of patients who did not perform preoperative regular sports. The differences found not only proved to be statistically significant, but also seem to be clinically relevant according to recent literature on the minimal clinical important difference. [16]

A lumbar disc herniation prompting surgery may be an incisive event in a patient’s everyday life. The patient’s recovery and improvement of pain after lumbar spine surgery may be influenced by various factors. [4]

The presence of depression might have a negative effect of the improvement after surgery. [17] Patients with mental disorders were excluded from our trial. Thus, BDI scores did not differ between the two investigated groups and this could be ruled out as a confounding factor. Furthermore, gender plays an important role in patients with radiculopathy caused by a lumbar disc herniation, as female gender might influence the patient’s pain perception in a negative way. The distribution of sexes was equal between our two study groups. Additionally, the surgical technique itself may be crucial for the patient’s outcome after surgery. Sequestrectomy as the standard surgical technique in our department and invariably used in this trial previously demonstrated superior results than lumbar discectomy. Lower recurrent back pain and superior satisfactory rates were observed in the short- as well as long-term follow-up. [3, 18]

Nevertheless, the influence of regular preoperative physical activity on postoperative outcome has not yet been investigated prospectively. Few studies could show that physiotherapy improves the patients’ reported outcome and healthcare consumption after surgery. [19] In general, there is evidence that a higher amount of physical activity is associated with less low back pain. [20] Endurance exercise for example, as preferred sports in our trial, is known to promote antinociceptive effects more likely on central processing rather than on peripheral pain. [21] It has to be kept in mind, however, that not only physical activity prior to surgery but as well thereafter may have an impact on surgical outcome. This can be within a rehabilitation program or by resuming preoperative sports. While postoperative rehabilitation did not differ between groups in our study, it is likely that preoperatively active patients resumed their activity postoperatively, which may be even more important for their respective outcome. Although sports on a regular basis seem to reduce pain perception, it does not decrease the risk to develop a lumbar disc herniation as the disc degeneration itself is multifaceted and traditionally attributed to age, mechanical loading, gender, trauma, and other factors impairing disc nutrition. [22, 23] Furthermore, a higher prevalence of structural abnormalities was reported in adolescent athletes compared to adolescent non-athletes, whereas an association to disc degeneration was not found. [24] Disc degeneration grades in our study population were equally distributed in the two investigated groups. On the contrary, Modic changes were less in the sports group. It could be speculated that regular physical activity may have a positive effect on disc and endplate nutrition resulting in less Modic changes in contrast to the mentioned negative effect of excessive exercise in adolescent athletes. The impact of Modic changes on postoperative clinical outcome, especially low back pain, however, is still a matter of debate, although an association with patient satisfaction has been reported. [25] These radiological differences between groups might explain some of the differences in clinical outcome.

In summary, regular exercise may positively influence regeneration of a disc degeneration early in the degenerative cascade by enabling a better disc nutrition. [26] It may be important that our patients were no professional athletes, as over-exercise may accelerate disc degeneration. [24]

Obviously, the treating surgeon cannot influence the patients’ baseline characteristics, namely preoperative physical activity and preoperative sports will not have an impact on surgical indications. Nevertheless, our results indicate that resumption or adoption of physical activity after disc surgery may be encouraged. Plus, the data support health promotion programs, which have gained popularity in the last decades to reduce health-related expenses by alleviating musculoskeletal pain. [27].

A recurrent lumbar disc herniation is the most prevalent cause for postoperative radicular pain. Rates quoted range from 3 to 19% with the higher rates usually in series with a longer follow-up. Multiple risk factors for recurrent disc herniations exist and are discussed controversially. Beneath young age, severe disc degeneration, traumatic events and gender seems to play a major role for the development of a recurrent disc herniation. [28] There was a trend towards higher reherniation rates in the NS group in our study. Large anular defects have been associated with higher reherniation rates [29] and technical nuances particularly concerning aggressiveness of disc removal also influence reherniation. [3, 18, 30] Our department’s standard operating procedure involves pure sequestrectomy in absence of a clear anular defect and limited discectomy in case of larger anular defects, so that there were no differences in surgical technique between groups. The sizes of the anular defects were not assessed in our study, but could have influenced the results. We postulate, however, that regular sports might even reduce the risk of recurrent disc herniations. Obviously, larger comparative studies are needed to confirm these findings.

Strengths of our study include the use of the validated, standardized comprehensive questionnaires like ODI, COMI or EQ-5D. Furthermore, we are able to present a homogenous study population, while we excluded individuals with major depression or chronic pain/neurological disorders. However, the small patient cohort is a limitation that might have introduced selection bias. The assessment of physical activity in our trial was based on self-reported values. This may have led to over-reporting of quality and quantity of physical activity. [31] Therefore, data would be more accurate when using activity measuring devices like an actigraph.

## Conclusion

Our data suggests that preoperative regular physical activity is an important influencing factor on the overall satisfaction and disability after lumbar disc surgery. It potentially has the capacity to reduce not only low back pain, but also leg pain after sequestrectomy. Therefore and because of many more health advantages humans may gain, physical activity should be an essential element in everyday’s life. The importance of sports may have been underestimated for surgical outcomes in general.
